# The Clinical Value of the Combined Detection of Enhanced CT, MRI, CEA, and CA199 in the Diagnosis of Rectal Cancer

**DOI:** 10.1155/2021/8585371

**Published:** 2021-07-13

**Authors:** Cuijuan Hao, Yanbin Sui, Jian Li, Yunxia Shi, Zhenxing Zou

**Affiliations:** Department of Medical Image, The Affiliated Yantai Yuhuangding Hospital of Qingdao University, Yantai 264000, China

## Abstract

**Background:**

To explore the clinical value of enhanced computed tomography (enhanced CT), magnetic resonance imaging (MRI), carcinoembryonic antigen (CEA), and cancer antigen 199 (CA199) in the diagnosis of rectal cancer (RC).

**Methods:**

A total of 156 patients with RC confirmed by postoperative pathology admitted to the Affiliated Yantai Yuhuangding Hospital of Qingdao University from March 2018 to November 2020 were included in the malignant group, and 52 patients with chronic proctitis in the benign control group. All patients underwent preoperative enhanced CT, MRI scans, and serum CEA and CA199 tests. The accuracy, sensitivity, and specificity of single and combined enhanced CT, MRI, CEA, and CA199 tests for the clinical staging of RC were calculated.

**Results:**

The postoperative pathological diagnosis showed that 35 cases of 156 RC patients were at *T*1 stage, 29 cases were at *T*2 stage, 24 cases were at *T*3 stage, 11 cases were at *T*4 stage, 23 cases were at *N*0 stage, 21 cases were at *N*1 stage, 8 cases were at *N*2 stage, 3 cases were at *M*0 stage, and 2 cases were at *M*1 stage. The positive rate of MRI in the diagnosis of RC was higher than that of enhanced CT. Serum CEA and CA199 levels in the malignant group were significantly increased compared with the benign group. The sensitivity, specificity, and accuracy of the combined detection were significantly higher than those of the single detection.

**Conclusion:**

Compared with enhanced CT, MRI has a higher detection rate of *T* and *N* stage in patients with RC. Combined enhanced CT, MRI, CEA, and CA199 can provide more accurate diagnosis and preoperative staging of RC patients.

## 1. Introduction

Rectal cancer (RC) is one of the common clinical malignant tumors, which occurs in the mucosa or submucosa. The surface of the RC tumor is uneven, the texture is generally hard, and the growth rate is fast. RC has a high incidence and metastasis rate, which seriously threatens the health of patients and affects the quality of life [1, 2]. However, the early symptoms of RC are not obvious, and some RC patients have entered the advanced stage when seeking treatment and missed the best treatment opportunity, resulting in poor clinical efficacy [3]. The commonly used methods for the treatment of RC are surgical resection and adjuvant chemotherapy, local resection, and endoscopic treatment [4, 5]. Clinical results show that the treatment and prognosis of patients with RC are closely related to the preoperative staging, and the more accurate the preoperative staging judgment is, the more reasonable the treatment plan can be selected by physicians [6]. Therefore, accurate preoperative staging of RC is the key to the prognosis of patients and the formulation of the best treatment plan.

Studies have reported [7, 8] that imaging examinations, such as MSCT and MRI, have outstanding value in the diagnosis of preoperative staging of RC. At present, enhanced CT scan has been used more and more widely in clinical practice. Its advantages include high image definition and fast scanning speed, which can make a more effective judgment on the location, size, and degree of invasion of the tumor and can also clearly show the metastasis of distant organs [9]. Routine magnetic resonance imaging has been widely used in preoperative staging of RC, but its accuracy and imaging characteristics have not been accurately determined [10]. In recent years, studies have found that tumor markers [11] play an important role in the occurrence and development of tumors. Oncoembryonic antigen (CEA) and carbohydrate antigen 19-9 (CA19-9) have been widely used in the diagnosis and prognosis follow-up of RC. But the sensitivity and specificity of the above indicators for single detection of RC are low [12–14]. Therefore, this study aims to explore the effectiveness of MSCT and MRI in the clinical staging of rectal cancer, and the clinical value of enhanced CT, MRI, CEA, and CA199 combined detection in the diagnosis of RC.

## 2. Materials and Methods

### 2.1. Study Design

A total of 156 patients with RC confirmed by postoperative pathology admitted to the Affiliated Yantai Yuhuangding Hospital of Qingdao University from March 2018 to December 2020 were included in this study. There were 95 males and 61 females. The average age was 52.5 ± 11.5 years from 27 to 69 years. There were 9 cases of highly differentiated adenocarcinoma, 124 cases of moderately differentiated adenocarcinoma, and 23 cases of poorly differentiated adenocarcinoma. There were 89 cases of middle and upper RC, and 67 cases of lower RC. The tumor diameter ranged from 1.5 to 7.7 cm, with an average of 4.9 cm. 52 patients with chronic proctitis were selected as the benign control group. Our study was approved by the medical ethics committee of the Affiliated Yantai Yuhuangding Hospital of Qingdao University.

Inclusion criteria: patients with RC met the relevant diagnostic criteria in Internal Medicine [15]; the patients and their family members provide informed consent, and all patients had no history of pelvic surgery and had not received pelvic radiotherapy or chemotherapy; also CT images, MRI images, and pathological data were clear and complete.

Exclusion criteria: patients with allergies to iodine contrast agents; patients with contraindications to MRI; patients with other benign and malignant tumors; patients with contraindications to examinations such as cardiac pacemakers and aneurysm clips.

### 2.2. Preparation before Inspection

All patients took liquid food two days before the examination, to avoid excessive feces accumulation in the body, which would affect the image quality. The day before the examination, patients took Senna granules (Z10910006, Yangzhou Xingdou Pharmaceutical Co., Ltd.) 10 g/time, twice/d, to clean the intestines. Eight hours *h* before the examination, patients were given a normal saline enema to maintain their intestinal cleanliness. One hour before the examination, the patients were given an intramuscular injection of 10 mg Racemic Anisodamine Hydrochloride Injection (H32024750, Xuzhou Lian Pharmaceutical Co., Ltd.) and drank 1000 ml water to make the bladder fully filled.

### 2.3. Enhanced CT Scanning

GE Light Speed 64-slice CT scanner was used for enhanced CT. Patients were placed in the left decubitus position and were supine after 800 ml of air was injected through the anus. After the plain scanning, patients were injected with 80 mL nonionic iodine contrast agent (3.5 mL/s). Dynamic enhanced CT scanning was performed at the intravenous phase (70 s after injection), arterial phase (30 s after injection), and balance phase (240 s after injection). After the scan, the data was transmitted to the CT postprocessing workstation.

### 2.4. MRI Scanning

Patients were placed in supine position and scanned the whole pelvic cavity. The scanning sequence was as follows: the sagittal T2WI sequence images were first scanned to observe the tumor size, scope, and distance from the tumor to the anus. Then, the axial high-resolution T2WI, T1WI sequence images perpendicular to the tumor segment, and the coronal T2WI parallel to the tumor segment were scanned. Finally, the DWI sequence images perpendicular to the tumor segment were scanned. After the plain scanning, sagittal, coronal, and cross-sectional enhanced scanning were performed. The total scanning time was controlled within 30 minutes. The data were transferred to the image processing workstation, and two physicians performed the staging diagnosis of the included patients.

### 2.5. CEA and CA199 Detection

5 mL of fasting blood from the cubital vein of the patients was collected and centrifuged at 3000 r/min for 5 min. The levels of serum CEA and CA19-9 were detected by an automatic chemiluminescence immunoanalyzer (CENTAUR XP, Siemens, Germany). The operation process was strictly in accordance with the manufacturer's instructions.

### 2.6. HE Staining

After fixation and dehydration, the specimens were embedded in paraffin and sectioned with a thickness of 4 *μ*m. The specimens were stained by hematoxylin-eosin staining [15] and observed under a microscope.

### 2.7. Evaluation Standard

Clinical staging of RC was determined according to the Updated Interpretation of the American Society of Oncology Colorectal Cancer Staging System [16]. Criteria for positive CEA and CA199: CEA > 5 ng/mL, CA199 > 37 U/mL. The sensitivity, specificity, and accuracy of enhanced CT, MRI, CEA, and CA199 alone and combined in the diagnosis of RC were compared and analyzed. Receiver operating curve (ROC) was used to calculate the diagnostic efficacy of CEA and CA19-9 in the diagnosis of RC. The positive criteria for the combined test: two or more positive results of enhanced CT, MRI, CEA, and CA199 tests.

### 2.8. Statistical Analysis

The data was analyzed by SPSS 23.0. Measurement data were expressed as mean ± SD, and enumeration data were expressed as number (%). *P* < 0.05 indicated that the difference was statistically significant.

## 3. Results

### 3.1. Postoperative TNM Staging Pathological Results of Rectal Cancer Patients

**Figure 1:**
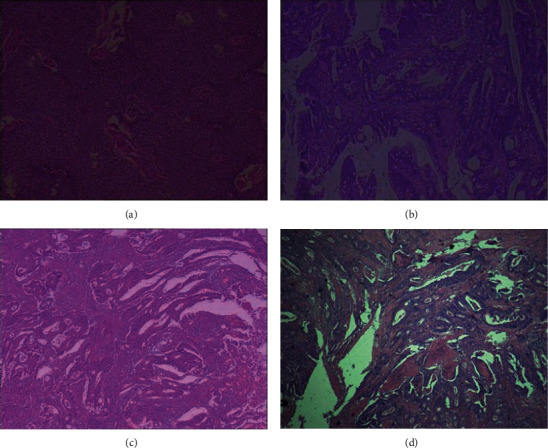
TNM postoperative pathological staging images of RC patients. (a) The image of raised, moderately differentiated squamous cell carcinoma (size: 2 × 2 × 1 cm). (b) The image of moderately differentiated ulcerative adenocarcinoma (size: 4.5 × 3.5 × 0.5 cm). (c) The image of moderately differentiated ulcerative adenocarcinoma (size: 4 × 3.5 × 2 cm). (d) The image of moderately differentiated ulcerative adenocarcinoma (size: 4.5 × 3.5 × 0.3 cm).

As shown in Figure 1, HE staining was performed on the specimens of patients with RC. The results displayed that 35 cases were *T*1 stage, 29 cases were *T*2 stage, 24 cases were *T*3 stage, 11 cases were *T*4 stage (Table 1), 23 cases were *N*0 stage, 21 cases were *N*1 stage, 8 cases were *N*2 stage (Table 2), and 3 cases were *M*0 stage, 2 cases of *M*1 stage (Table 3).

### 3.2. Contrast of the Diagnostic Results of Enhanced CT and MRI in Preoperative TNM Staging of RC Patients

**Figure 2:**
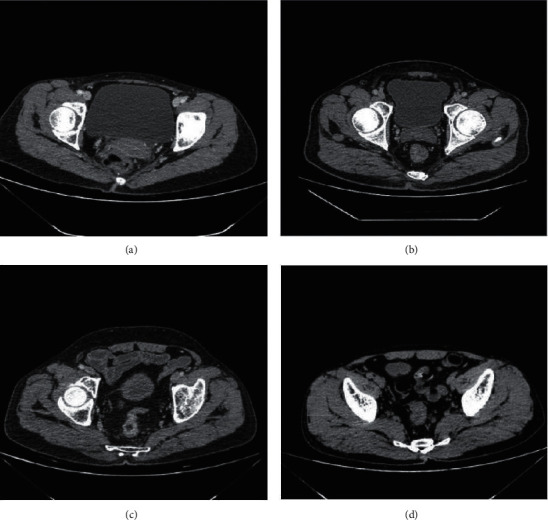
Enhanced CT image of patients with RC. (a) A 55-year-old woman in stage *T*1 RC presented with slight enhancement in submucosal lesions. (b) A 49-year-old man in stage *T*3 RC with involvement of the muscularis propria and perirectal tissues. (c) A 61-year-old female patient in stage *N*1 RC had subserosal invasion and lymph node metastasis. (d) A 37-year-old woman in stage *N*2 RC with invasion to the muscularis propria and lymph node metastasis.

**Figure 3:**
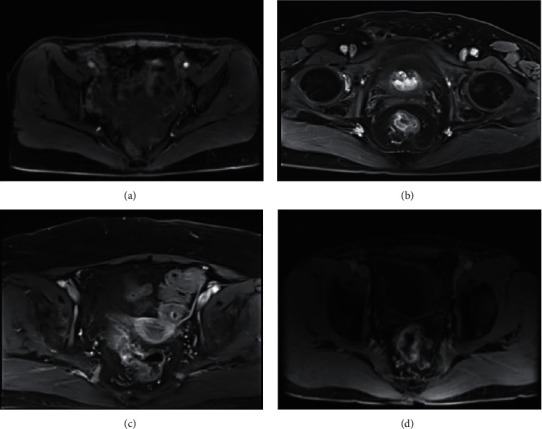
MRI image of patients with RC. (a) The patient was in stage *T*1 RC with high signal submucosa below the lesion, and without involvement of muscularis propria. (b) The patient was in stage *T*3 RC with muscularis propria and perirectal adipose tissue, but not mesocrectum and fascia. (c) The patient was in stage *N*1 RC, and with lymph node metastasis. (d) The patient was in stage *N*2 RC with bilateral lymph node metastasis.

According to enhanced CT and MRI image data, the preoperative TNM staging of RC patients was diagnosed (Figures 2(a)–2(d) and 3(a)–3(d)). The detection rate of MRI for *T* and *N* staging was higher than that of enhanced CT, and the detection rate of *M* staging was consistent. Therefore, MRI has a higher positive rate than enhanced CT in the diagnosis of RC (Tables 1–3).

### 3.3. Comparison of Serum CEA and CA199 Levels between the Two Groups

We detected the levels of serum CEA and CA199 in malignant group and benign group. The results displayed that serum CEA and CA199 levels in malignant group were significantly increased compared with benign group (*P* < 0.001, Table 4).

### 3.4. ROC Curve of CA199 and CEA in the Diagnosis of RC

**Figure 4:**
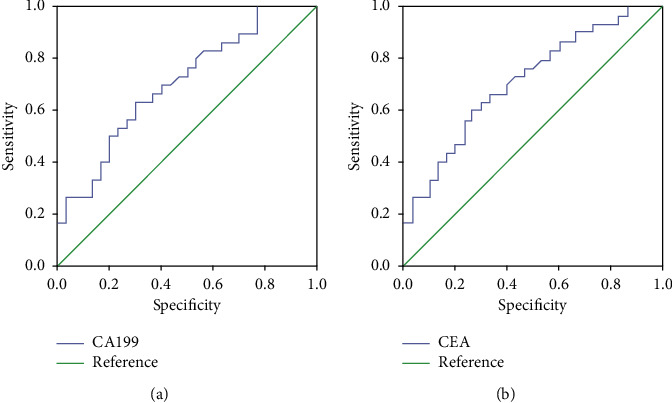
ROC curve of CA199 and CEA in diagnosis of RC. (a) CA199 curve of CEA in diagnosis of RC. (b) ROC curve of CEA in diagnosis of RC.

ROC curve indicated that the AUC was 0.713 (0.584–0.843), and Youden index was 0.331 for CEA diagnosis of RC (Figure 4(a)). The AUC of CA199 in the diagnosis of RC was 0.706 (0.575–0.836), and the Youden index was 0.274 (Figure 4(b)).

### 3.5. Comparison of Single and Combined Tests of Enhanced CT, MRI, CEA, and CA199 in the Diagnosis of RC

The sensitivity, specificity, and accuracy of enhanced CT in the diagnosis of RC were 73.08%, 78.85%, and 74.52%, respectively. The sensitivity, specificity, and accuracy of MRI in the diagnosis of RC were 83.97%, 86.54%, and 84.62%, respectively. The sensitivity, specificity, and accuracy of CA199 in the diagnosis of RC were 51.92%, 78.85%, and 58.65%, respectively. The sensitivity, specificity, and accuracy of CEA in the diagnosis of RC were 57.69%, 86.92%, and 62.50%, respectively. The sensitivity, specificity, and accuracy of combined detection in the diagnosis of RC were 94.23%, 98.08%, and 95.19%, respectively. As shown in Table 5, the sensitivity, specificity, and accuracy of combined diagnosis were significantly higher than those of a single diagnosis (*P* < 0.05, Table 5).

## 4. Discussion

At present, the incidence of RC in China is increasingly high, and it is more common in middle-aged and elderly groups [17]. Patients with early RC have no specific clinical manifestations and are often diagnosed in the middle and advanced stages, with unsatisfactory treatment and prognosis [3]. Therefore, it is of great significance for the early diagnosis of RC patients and the accurate judgment of postoperative staging.

Enhanced CT and MRI are widely used in preoperative staging detection [18, 19]. Enhanced CT has advantages of convenient operation, high spatial resolution, and good effect in the detection of distant metastasis [20]. Enhanced CT can also carry out a comprehensive scan of the tumor size, size, and infiltration in RC patients, and then quickly obtain comprehensive and detailed images [21]. Enhanced CT can be used to observe images through multiplanar recombination, thus obtaining images of any section of the patient's body. In addition, the diagnosis can help physicians have a deeper understanding of the patient's symptoms, the details of tumor lesions and the relationship between the patient's internal space anatomy, so as to improve the accuracy of malignant tumor staging [22, 23]. However, as the infiltration of RC in the early intestinal wall is not significant, and enhanced CT is difficult to stratify the patient's intestinal wall, preoperative staging diagnosis often lacks good accuracy, especially for the detection of early RC [24]. MRI can distinguish the three-layer structure of intestinal wall and the adjacent fat background rectal fascia, which is suitable for the benign and malignant differentiation and accurate staging of tumors [25, 26]. MRI can conduct comprehensive detection of tumor properties, layers of structure, lymph node metastasis, and organ infiltration in the patient's body and directly obtain comprehensive image information by skipping image and information reconstruction [27, 28]. Moreover, MRI also has multiple sequence imaging and a variety of image types, which can carry out in-depth and comprehensive detailed detection of rectum, bladder, vagina, and other areas of RC lesions. In this respect, the detection performance of MRI is significantly better than that of enhanced CT [29], which is consistent with the results of this study.

Studies have shown that CEA and CA199 play an important role in the diagnosis, prognosis, and recurrence monitoring of RC [30]. CEA, a high-molecular weight glycoprotein produced by normal colon cells, acts as an intercellular adhesion molecule and can promote the aggregation of RC cells. CEA is one of the most widely used tumor markers in RC [31]. However, due to the poor sensitivity and specificity of CEA, RC patients cannot be completely diagnosed by CEA content detection alone [32]. CA199 can be used as one of the common tumor markers of RC [33]. However, the sensitivity and accuracy of these two indicators in single detection of RC are poor, and the preoperative screening value is not high. However, it is helpful for early clinical detection of suspected RC patients and further combined with auxiliary detection methods such as preoperative imaging to improve the accuracy of preoperative staging of RC.

In this study, by comparison and analysis of examination results and postoperative pathological staging results, the sensitivity, specificity, and accuracy of MRI in the detection of preoperative TN staging of RC patients were significantly better than those of enhanced CT detection, and the obtained results were consistent with those of the above research reports [28]. The levels of CEA and CA199 in the malignant group were higher than those in the benign group, indicating that the levels of both indexes increased with the deepening of the malignant tumor. The sensitivity, specificity, and accuracy of the combined test for the preoperative staging of RC patients were significantly higher than those of the single test. It is suggested that the combined detection can make advantages complement each other and have higher diagnostic value and can provide data support for a more accurate diagnosis of RC.

In conclusion, the combined detection of enhanced CT, MRI, CEA, and CA199 levels can improve the detection rate of RC and make a more accurate judgment of preoperative staging. It has high diagnostic value and can provide the clinical basis for early diagnosis and treatment of RC patients, which is worthy of promotion and application. However, the sample size of this study was small, and the combined diagnosis in the clinical diagnosis of RC still needs further study.

## Figures and Tables

**Table 1:** Enhanced CT and MRI in the preoperative *T* staging of RC patients.

Item	Clinical staging	*T*1	*T*2	*T*3	*T*4	Total
Enhanced CT	*T*1	21	7	0	0	28
	*T*2	11	20	3	0	34
	*T*3	3	2	18	2	25
	*T*4	0	0	3	9	12

MRI	*T*1	26	3	0	0	29
	*T*2	8	23	3	0	34
	*T*3	1	3	20	1	25
	*T*4	0	0	1	10	11

Total		35	29	24	11	99

**Table 2:** Enhanced CT and MRI in the preoperative *N* staging of RC patients.

Item	Clinical staging	*N*0	*N*1	*N*2	Total
Enhanced CT	*N*0	17	3	0	20
	*N*1	6	17	1	24
	*N*2	0	1	7	8

MRI	*N*0	19	1	0	20
	*N*1	4	20	0	24
	*N*2	0	0	8	8

Total		23	21	8	52

**Table 3:** Enhanced CT and MRI in the preoperative *M* staging of RC patients.

Item	Clinical staging	*M*0	*M*1	Total
Enhanced CT	*M*0	3	0	3
	*M*1	0	2	2

MRI	*M*0	3	0	3
	*M*1	0	2	2

Total		3	2	5

**Table 4:** Comparison of serum CEA and CA199 levels between the two groups ($\bar{x}$ ± *s*).

Group	*n*	CEA (ng/mL)	CA199 (U/mL)
Malignant group	156	5.63 ± 1.02	39.58 ± 3.47
Benign group	52	1.85 ± 0.64	12.33 ± 1.52
*X* ^2^		21.462	43.078
*P* value		<0.001	<0.001

**Table 5:** Comparison of single and combined enhanced CT, MRI, CEA, and CA199 in the diagnosis of RC (%).

Item	Sensitivity	Specificity	Accuracy	Positive prediction rate	Negative prediction rate
Enhanced CT	73.08 (114/156)	78.85 (41/52)	74.52 (155/208)	91.20 (114/125)	49.40 (41/83)
MRI	83.97 (131/156)	86.54 (45/52)	84.62 (176/208)	94.93 (131/138)	64.29 (45/70)
CA199	51.92 (81/156)	78.85 (41/52)	58.65 (122/208)	88.04 (81/92)	35.34 (41/116)
CEA	57.69 (90/156)	86.92 (40/52)	62.50 (130/208)	88.24 (90/102)	37.74 (40/106)
Combined diagnosis	94.23 (147/156)	98.08 (51/52)	95.19 (198/208)	99.32 (147/148)	85.00 (51/60)

## Data Availability

The data used to support the findings of this study are available from the corresponding author upon request.
